# Supplementary material for: Mir-132/212 is required for maturation of binocular matching of orientation preference and depth perception
Source: Nat Commun. 2017 May 23;8:15488. doi: 10.1038/ncomms15488 (PMC5457514; doi:10.1038/ncomms15488)
Supplement: Supplementary Information — Supplementary Figures. [file ncomms15488-s1.pdf]

Supplementary Figure 1.

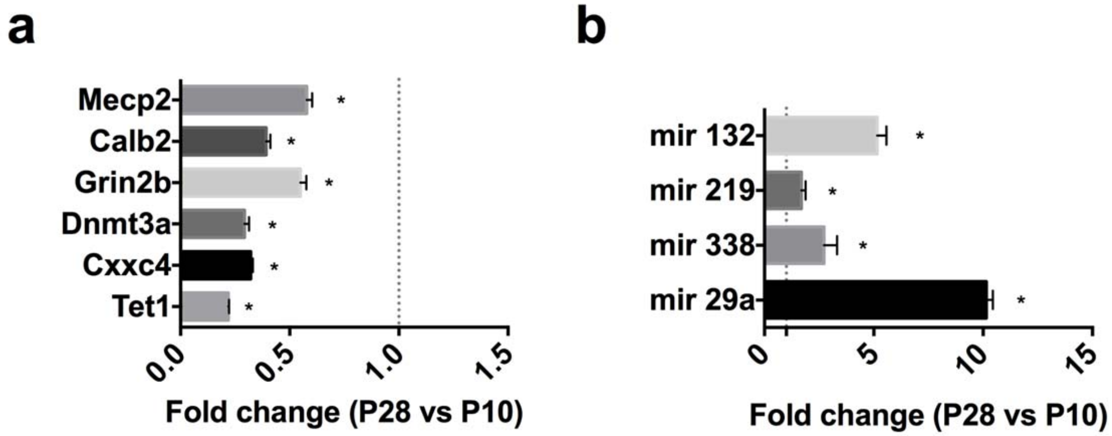

Supplementary Fig. 1. RT-PCR validation of RNAseq and small RNAseq data. mRNAs and miRNAs shown to be age regulated by RNA and small RNA sequencing showed the same regulation when assessed by RT-PCR. The data from P10 and P28 visual cortex samples were analyzed using the ddCT methods. **(A)** Fold change in P28 vs. P10 mice; Tet1, Cxxc4, Dnmt3a, Grin2b, Calb2, Mecp2 (t test, p < 0.0001; n =4 for each group and target). **(B)** Fold change in P28 vs. P10 mice for miR-29a, miR-338, miR-219, miR-132 (t test, p < 0.02; n = 4 for each group and target). Error bars represent SEM. Asterisks indicate statistical significance (p<0.05).

Supplementary Figure 2.

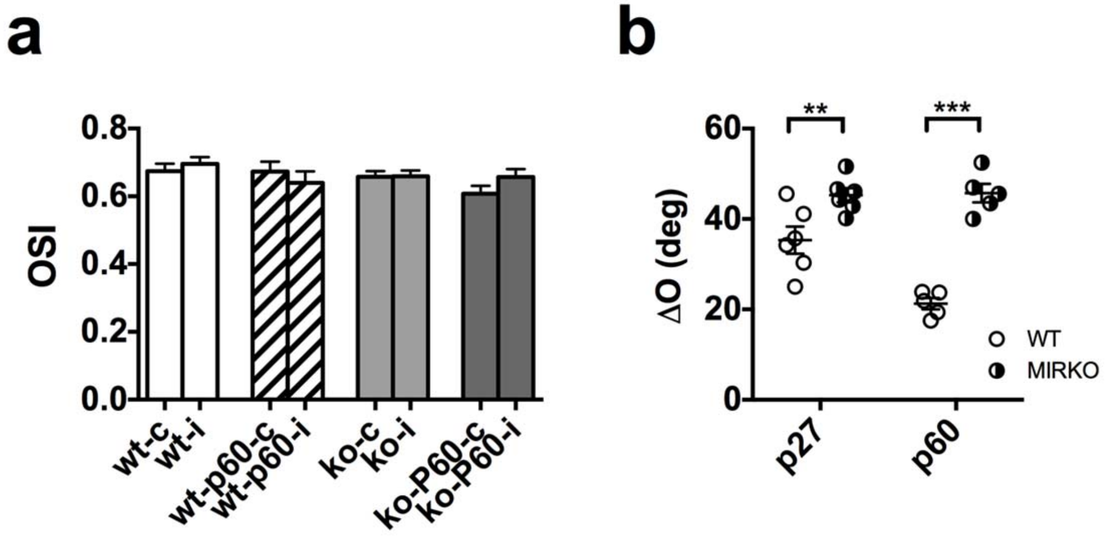

Supplementary Fig 2. (A) OSI in wt and mutant mice (ko) at P30 and P60 (wt-P60, ko-P60). Error bars represent SEM. **(B)** ΔO values for wt, ko, wt-P60 and ko-P60 animals. Results are analyzed by case. Whiskers represent the average ΔO ± SEM for each experimental group. Circles represent individual ΔOs for each animal. Binocular matching is strongly impaired in miR-132/212 null mice both at P27-28 and P60 (Two-way ANOVA, effect of genotype $p < 0.001$; post hoc Holm-Sidak test, wt vs. ko: P27-28 $p < 0.01$, P60 $p < 0.001$). ** $p<0.01$, *** $p<0.001$.

Supplementary Figure 3.

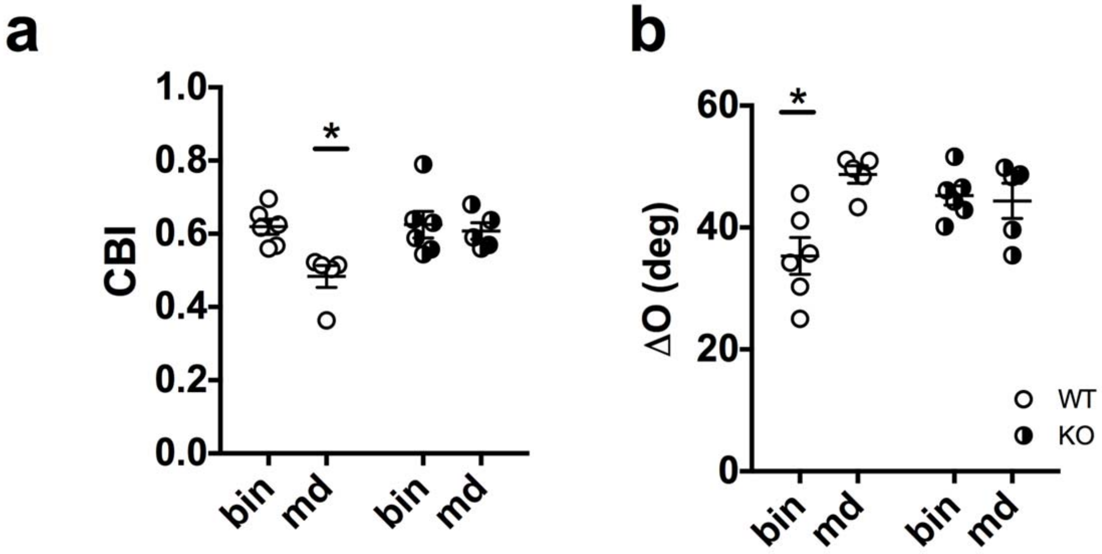

Supplementary Fig 3. (A) Contralateral bias index (CBI) values for wt and ko mice monocularly deprived (md) or nondeprived (bin). Whiskers represent the average CBI ± SEM for each experimental group; circles represent individual CBIs for each animal. CBI of null mice was not significantly different from that of wt animals (Two-way ANOVA, genotype x condition interaction $p < 0.05$; post-hoc Holm-Sidak test, $p = 0.96$) and from that of md null mice (post-hoc Holm-Sidak test $p = 0.97$), whereas md-wt mice displayed the typical ocular dominance (OD) shift towards the open eye (post-hoc Holm-Sidak test, $p < 0.05$). **(B)** ΔO values for wt, ko, wt-md and ko-md animals. Whiskers represent the average ΔO ± SEM for each experimental group; circles represent individual ΔO for each animal. The effect of MD on binocular matching is occluded by the deletion of miR-132/212 (Two-way ANOVA, genotype x condition interaction $p < 0.01$; post-hoc Holm-Sidak test: wt vs wt-md $p < 0.01$, ko vs. ko-md $p = 0.795$). * $p<0.05$.

Supplementary Figure 4.

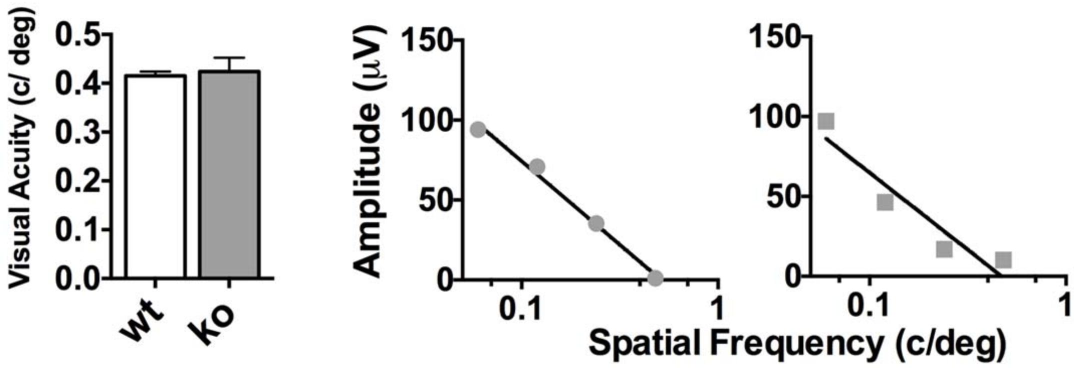

Supplementary Fig. 4 *miR132/212* deletion does not affect visual acuity. Histograms represent the average VA ± SEM for wild-type (n = 3) and *miR132/212* null mice (n = 3). Error bars represent SEM. Examples of VEP curves for single mice are reported on the right.

Supplementary Figure 5

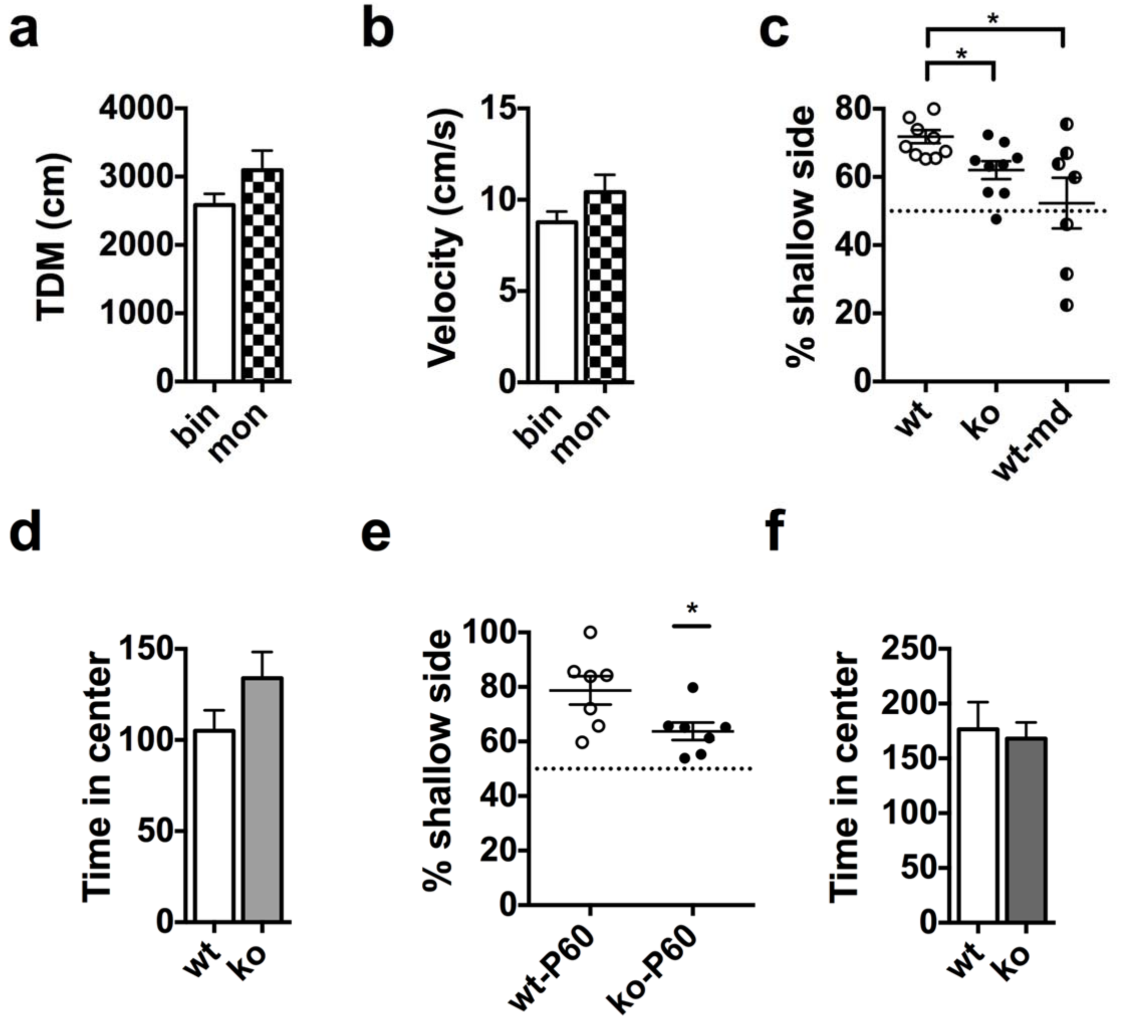

Supplementary Fig. 5. (A) Total distance moved (TDM) and **(B)** velocity in bin and mon mice. **(C)** Average time spent in the shallow side for wt, ko and wt-md animals. Whiskers represent the average % in shallow side ± SEM for each experimental group; circles represent individual percentages for each animal. Dotted line represents chance level. **(D)** No difference was detected between wt and ko animals in the time spent in the center of the visual cliff arena at P30 (t-test, p = 0.126). **(E)** Average time spent in the shallow side for wt-P60 and ko P60 mice. Whiskers represent the average % in shallow side ± SEM for each experimental group; circles report individual percentages for each animal. **(F)** Exploration time of the center of

visual cliff arena is totally comparable in wt-P60 and ko-P60 mice (t-test, $p = 0.767$). * $p < 0.05$.

Supplementary Figure 6.

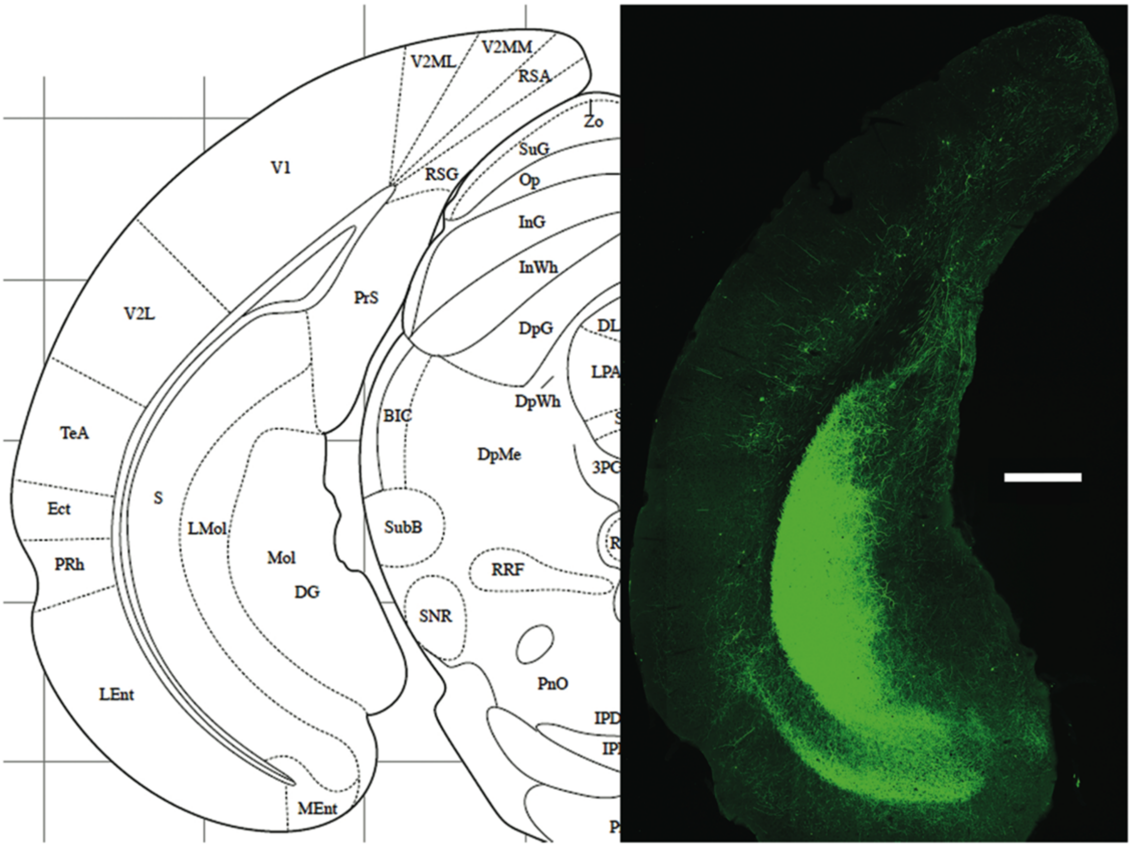

Supplementary Fig. 6 Low magnification image of the visual cortex of a *miR132/212* mice crossed with GFP expressing line M mice**.** Please note GFP positive layer V pyramidal cell bodies and dendrites in the visual cortex. Left hand side: scheme with anatomical location of V1 (from Paxinos G and Franklin KBJ The mouse brain in stereotaxic coordinates 2nd edition 2001, Academic Press) Scale bar 500 microns

Supplementary Figure 7.

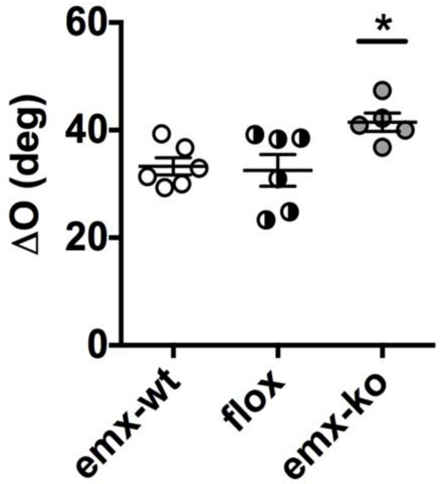

Supplementary Fig. 7 ΔO values for emx-wt, flox, and emx-ko animals. Whiskers represent the average ΔO ± SEM for each experimental group; circles represent individual ΔOs for each animal. Binocular matching is significantly impaired in emx-ko mice (One-way ANOVA $p < 0.05$, post hoc Holm-Sidak test, miR-132/212$^{fl/fl}$ vs Emx1:Cre-miR-132/212$^{-/-}$ $p < 0.05$; Emx1:Cre-wt vs Emx1:Cre-miR-132/212$^{-/-}$ $p < 0.05$; Emx1:Cre-wt vs miR-132/212$^{fl/fl}$ $p = 0.808$) * $p<0.05$.
